# The Promise of Lung Organoids for Growth and Investigation of *Pneumocystis* Species

**DOI:** 10.3389/ffunb.2021.740845

**Published:** 2021-09-01

**Authors:** Nikeya Tisdale-Macioce, Jenna Green, Anne-Karina T. Perl, Alan Ashbaugh, Nathan P. Wiederhold, Thomas F. Patterson, Melanie T. Cushion

**Affiliations:** ^1^Department of Internal Medicine, University of Cincinnati College of Medicine, Cincinnati, OH, United States; ^2^Medical Research Service, Cincinnati Veterans Affairs Medical Center, Cincinnati, OH, United States; ^3^Department of Pediatrics, University of Cincinnati College of Medicine, Cincinnati, OH, United States; ^4^Perinatal and Pulmonary Biology, Cincinnati Children's Hospital Medical Center, The Perinatal Institute and Section of Neonatology, Cincinnati, OH, United States; ^5^Department of Pathology, The University of Texas Health Science Center, San Antonio, TX, United States; ^6^Department of Medicine, The University of Texas Health Science Center, San Antonio, TX, United States; ^7^Section of Infectious Diseases, South Texas Veterans Health Care System, San Antonio, TX, United States

**Keywords:** *Pneumocystis* pneumonia, lung organoids, opportunistic fungal pathogen, immunosuppressed hosts, *Pneumocytis* species

## Abstract

*Pneumocystis* species (spp.) are host-obligate fungal parasites that colonize and propagate almost exclusively in the alveolar lumen within the lungs of mammals where they can cause a lethal pneumonia. The emergence of this pneumonia in non-HIV infected persons caused by *Pneumocystis jirovecii* (PjP), illustrates the continued importance of and the need to understand its associated pathologies and to develop new therapies and preventative strategies. In the proposed life cycle, *Pneumocystis* spp. attach to alveolar type 1 epithelial cells (AEC1) and prevent gas exchange. This process among other mechanisms of *Pneumocystis* spp. pathogenesis is challenging to observe in real time due to the absence of a continuous *ex vivo* or *in vitro* culture system. The study presented here provides a proof-of-concept for the development of murine lung organoids that mimic the lung alveolar sacs expressing alveolar epithelial type 1 cells (AEC1) and alveolar type 2 epithelial cells (AEC2). Use of these 3-dimensional organoids should facilitate studies of a multitude of unanswered questions and serve as an improved means to screen new anti- PjP agents.

## Introduction

*Pneumocystis* spp. are host-obligate fungal parasites that colonize and propagate within mammalian lungs. The species infecting humans, *Pneumocystis jirovecii*, causes pneumonia (PjP) in individuals that are immunosuppressed. PjP was prevalent during the HIV/AIDs epidemic (Mills, 1986), but today there are more hospitalized patients with PjP with underlying malignancies rather than HIV infection. In fact, PjP still threatens any individual with a weakened immune system (Ahn et al., 2016; Higashi et al., 2017; Redelman-Sidi et al., 2018). *Pneumocystis* spp. mostly reside in the mammalian lung and exhibit host-specificity, e.g., *P. jirovecii, P. carinii*, and *P. murina* will only infect humans, rats, and mice, respectively. While exceptions to this long-held concept are emerging (Babb-Biernacki et al., 2020; Cisse et al., 2021), these 3 species appear to adhere to this view. Efforts to understand the complete life cycle of these fungal pathogens are complicated because a continuous *ex vivo* or *in vitro* culture is required to support and sustain growth and such a system remains elusive despite extensive efforts (Cushion et al., 2021). In this study, we present a *P. murina- Mus musculus* system which fulfills the species-specificity concept. The intent of this paper is to serve as a proof of principle examining the potential use of lung organoids to investigate many aspects of the biology of the Pneumocystis species. It is our intent to get this technology out to the scientific community to allow other labs to initiate studies using different approaches and applications, and thus improve the system.

Microscopic observations of infected mammalian lungs and genetic analyses led to the current proposed life cycle with at least three major morphological stages: trophic forms, pre-ascus intermediate forms, and asci (Hauser and Cushion, 2018). Trophic forms are highly irregular in shape and range from 1 to 5 μm in length. These forms appear to be haploid and are thought to mate via a primary homothallic sexual strategy, though there is much more to understand about this process (Richard et al., 2018; Luraschi et al., 2019). It has long been held that trophic forms replicate by binary fission, but more recent studies have brought this mode of replication into question (Cushion et al., 2010; Hauser and Cushion, 2018; Miesel et al., 2021). Trophic forms do attach to the alveolar epithelial type 1 cells (AEC1) (Shiota et al., 1986; Kottom et al., 2008) to the exclusion of alveolar epithelial type 2 cells (AEC2). After fusion and karyogamy, there is a gradual formation of the asci, which involves the deposition of β-1,3-D-glucan resulting in a mature ascus containing 8 spores (Cushion and Stringer, 2010). These intermediate forms range in size from ~4–8 μm (Cushion, 1998). The asci are considered essential for sexual replication (Cushion et al., 2018) and vital for transmission (Cushion et al., 2010). Genetic analyses of *Pneumocystis* spp. suggests that these fungi are obligate biotrophs, due to loss of genes necessary for amino acids and thiamine biosynthesis, the need to uptake inorganic nitrogen and sulfur, the catabolism of purines, loss of RNA interference machinery, lack of virulence characteristics, and the reductions of lytic proteases (Cushion et al., 2007; Cushion and Stringer, 2010; Hauser et al., 2010; Cisse et al., 2012, 2014).

Mammalian lungs are complex organs comprised of several different cell types. These cells are structurally arranged in a single tube formation at the proximal region to create the trachea, which is joined by forming branch-like tubes in the distal regions of the lungs, and these function to bring air into alveoli for gas exchange. Recently, lung organoids, *in vitro* three-dimensional structures supported by extracellular matrix, are becoming important research tools to elucidate lung development, function, and disease pathologies. Lung organoids maintain characteristics that closely resemble those of intact lungs including multiple cell types that are arranged in accurate structures and patterns and display similar cell-to-cell interactions that occur within the lungs (Nadkarni et al., 2016; Nikolić and Rawlins, 2017; Nikolić et al., 2017).

There are currently several types of lung organoids derived from primary cells: (1) bronchiolar organoids that are comprised of club, ciliated, and goblet cells (Lee et al., 2014); (2) bronchioalveolar organoids which are comprised of club, ciliated, goblet, AEC1 and AEC2 (Lee et al., 2014); (3) alveolospheres made of AEC1 and AEC2 cells (Barkauskas et al., 2013; Lee et al., 2014; Gokey et al., 2021); and (4) tracheo/bronchoshperes comprised of basal, ciliated and goblets cells (Rock et al., 2009). Collectively, these models represent the proximal, distal, and alveolar regions of the respiration system, which provide useful platforms for therapeutic and clinical applications (Barkauskas et al., 2017; Nikolić and Rawlins, 2017). In addition to therapeutic applications, organoids assist with elucidating human disease pathologies, such as cystic fibrosis, which were once limited, due to the lack of model systems that accurately mimicked human physiology (Ramani et al., 2018). Such has been the case with PjP. The lack of a 3-D system to understand this genus' pathology as well as other applications such as pre-clinical drug testing, together with the lack of any means to continuously propagate these fungi outside the lungs have resulted in significant gaps in knowledge regarding these important fungal pathogens (Cushion et al., 2021). The emerging field of lung organoids holds promise for growth of *Pneumocystis* spp. outside the host; assessment of pathologic processes; and drug screening in a 3-D model which contains the AEC1 cells to which these fungi attach. Here we report a proof of concept that lung organoids can potentially be used to further the study of *Pneumocystis* spp.

## Materials and Methods

### Mice

Six-12-week-old B6 wild-type mice were used for these experiments and purchased from Charles Rivers Laboratories (Raleigh, NC). All animal procedures were reviewed and approved by the Institutional Animal Care and Use Committee at the Cincinnati Veterans Administration Medical Center (Cincinnati, OH).

### Lung Digestion

All mice were humanely euthanized by an overdose of CO_2_. All steps were performed under aseptic conditions. After ensuring the mice were appropriately euthanized by toe pinch, the abdominal cavity was cut to expose and exsanguinate the abdominal aorta. The thoracic cavity was then opened to expose the lungs and the heart. The heart was perfused with 10 mL of cold sterile PBS through the right ventricle to flush out the blood in the lungs. The trachea was exposed and cannulated with a 24G cannula to instill 1 mL of 100 U dispase (Corning, Corning, NY) into the lungs. The trachea, heart and lungs were removed. Lung lobes were dissected from the trachea, heart, and connective tissue and incubated at room temperature for an additional 5–10 min in the dispase solution. Lungs were transferred to a C-tube (Miltenyi Biotec, Gaithersburg, MD) with the addition of 60 μL DNase (10 KU/mL) (Corning, Corning, NY), and homogenized with the GentleMACsDissociator program m_lung_1.01. Lung homogenate was incubated at 37°C for 10 min to assist with the breakdown of the lung tissue to achieve single cell suspension, centrifuged at 1,500 rpm at 4°C for 5 min. To lyse red blood cells, the lung homogenate was treated with 0.85% aqueous ammonium chloride, incubated for 5 min at 37°C, then pelleted at 1,500 rpm at 4°C for 5 min.

### Isolation of Primary Lung Epithelial and Fibroblast Cells

Lung epithelial cell isolation was initiated by using CD326 (EpCAM) MicroBeads Isolation Kit (Miltenyi Biotec, Gaithersburg, MD) following the vendor's protocol. Briefly, lung homogenate cell pellets were resuspended in 90 μL of kit buffer per 10^7^ total cells, to which 10 μL of CD326 (EpCAM) microbeads were added, mixed well, and incubated for 15 min at 4°C. Cells were then washed with 1–2 mLs of buffer, centrifuged at 300 × g for 10 min, and after aspiration of the supernatant, were resuspended up to 10^8^ cells in 500 μL buffer. Cell suspensions were applied to prepared LS columns and washed 3 times with 3 mL of buffer. The column was removed from the separator, 5 mL of buffer was placed onto column, and the magnetically labeled cells were then flushed into a 15 mL conical tube.

Lung fibroblasts were obtained by 2 methods: (1) the flow-through from CD326 (EpCAM) isolation described above was collected and plated in DMEM/F12, 15% FBS, 1% penicillin/streptomycin media, allowing fibroblast to adhere to the plate. Fibroblast were grown to confluence for either organoid development or frozen for future assays; (2) 2 weeks prior to isolating lung epithelial cells, mice were sacrificed, and lung digestions were performed as described above. Lung homogenates were plated to allow fibroblasts to adhere to the 10 mm plate (Corning, Corning, NY) (Seluanov et al., 2010).

### Organoid Development

To produce feeder cells, fibroblasts were treated with 10 ug/mL Mitomycin C (Sigma-Aldrich, St. Louis, MO) for at least 2 h to activate them. For organoid development, 30,000 EpCAM positive cells were mixed with 100–150,000 fibroblast feeder cells in 50–100% growth factor Matrigel (Trevigen, Gaithersburg, MD). Cell mixture plus Matrigel was either plated on 24-transwells (Corning, Corning, NY) or Nunc IVF 4 well dishes (ThermoFisher Scientific, Waltham, MA) for microinjection. For the 24-transwells, 500 μL MTEC/Plus media (see below) was added to the lower chamber. For the Nunc IVF 4 well dishes, 500 μL MTEC/Plus media was added in the chamber where the Matrigel with cells were plated. Multiple media changes occurred during the development of the organoids, approximately every 2–3 days. The composition of this medium included the following: MTEC Basic Media: DMEM-Ham's F-12 (1:1 v/v), 15 mM HEPES, 3.6 mM Sodium Bicarbonate, 4 mM L-glutamine, 5% FBS (Hyclone ThermoFisher, Omaha, NE), 100 U/ml penicillin (Fisher Scientific, Pittsburg, PA), 100 ug/ml streptomycin (Fisher Scientific, Pittsburg, PA). MTEC/Plus Media: MTEC Basic Media, 10 ug/ml insulin (Fisher Scientific, Pittsburg, PA), 5 ug/mL transferrin (Fisher Scientific, Pittsburg, PA), 0.1 ug/mL cholera toxin (Fisher Scientific, Pittsburg, PA), 25 ng/mL epidermal growth factor (Fisher Scientific, Pittsburg, PA), 30 ug/mL bovine pituitary extract (Fisher Scientific, Pittsburg, PA) (freshly added to media immediately prior to initiation of organoid growth) 12.5 ng/mL retinoic acid (Fisher Scientific, Pittsburg, PA), 50 ng/mL of Fibroblast growth factor (Fisher Scientific, Pittsburg, PA), and/or either/combination of 10 uM Rho-associated protein kinase inhibitor (Fisher Scientific, Pittsburg, PA) and 10 ng/mL Leukemia inhibitory factor (Life Technologies, Frederick, MD).

### Micro-Injection of Murine Lung Organoids With *P. murina*

At ~3–5 weeks post plating, organoids are large enough for micro-injections. Leica MZ16FA Stereo Zoom Microscope was used at a magnification of 60X or greater to facilitate injections. Ten to−30 nL of a *P. murina* suspension in PBS (1 × 10^6^-2 × 10^7)^) was injected per organoid using a Drummond Nanoject II microinjector. Approximately 100–6,000 organisms were injected into each organoid.

### Histological Evaluation of Murine Organoids

Whole mount Matrigel™ containing organoids were washed twice with cold PBS, fixed with 4% paraformaldehyde, followed by 2 washes with cold PBS. For paraffin sections, the fixed whole mount Matrigel™ was removed from inserts or plates, embedded in paraffin, and cut into 5–6 uM sections. Paraffin sections were deparaffinized, rehydrated, immersed in citrate buffer for antigen retrieval (95–99°C) for 45 min in a steamer, allowed to cool down for 10 min with cool running tap water, immersed in PBS with 0.25% Triton X for 10 minutes, and blocked with BlockAid (Invitrogen ThermoFisher, Waltham, MA) for 1 hour at room temperature. Primary antibodies were diluted and incubated over night at 4°C or incubated for 2–3 h at 37°C. Primary antibodies were 1:500 rabbit anti-aquaporin-5 (AQP5) (Abcam, Cambridge, MA), 1:500 rabbit anti- SFTPC (Abcam, Cambridge, MA), 1:00 rabbit anti- MSG (Sunkin et al., 1998). Secondary antibodies were conjugated with Alexa Fluro – 488, 594, 647 and placed on sections after washing away excess primary antibodies with PBS. Secondary antibodies were diluted 1:500, incubated for 1–2 h at 37°C, excess secondary antibodies were washed away with PBS, and 1:1000 dilution of DAPI was used to stain the nuclei. The final staining resulted in the combination of rabbit anti-aquaporin-5 paired with Alexa Fluro 488, rabbit anti-SFTPC paired with Alexa Fluro 647, and rabbit anti-MSG paired with Alexa Fluro 594. For paraffin-stained sections, anti-fade mounting media and cover slips were used to preserved samples.

### Imaging of Organoids

Whole mount Matrigel™ with organoids were observed with the Lecia MZ16FA Stereo Zoom Microscope. Immunofluorescent images were captured with a Zeiss LSM710 Live Duo Confocal Microscope.

## Results and Discussion

### Establishing Murine Lung Organoids With Type 1 Alveolar Epithelial Cells

**Figure 1:**
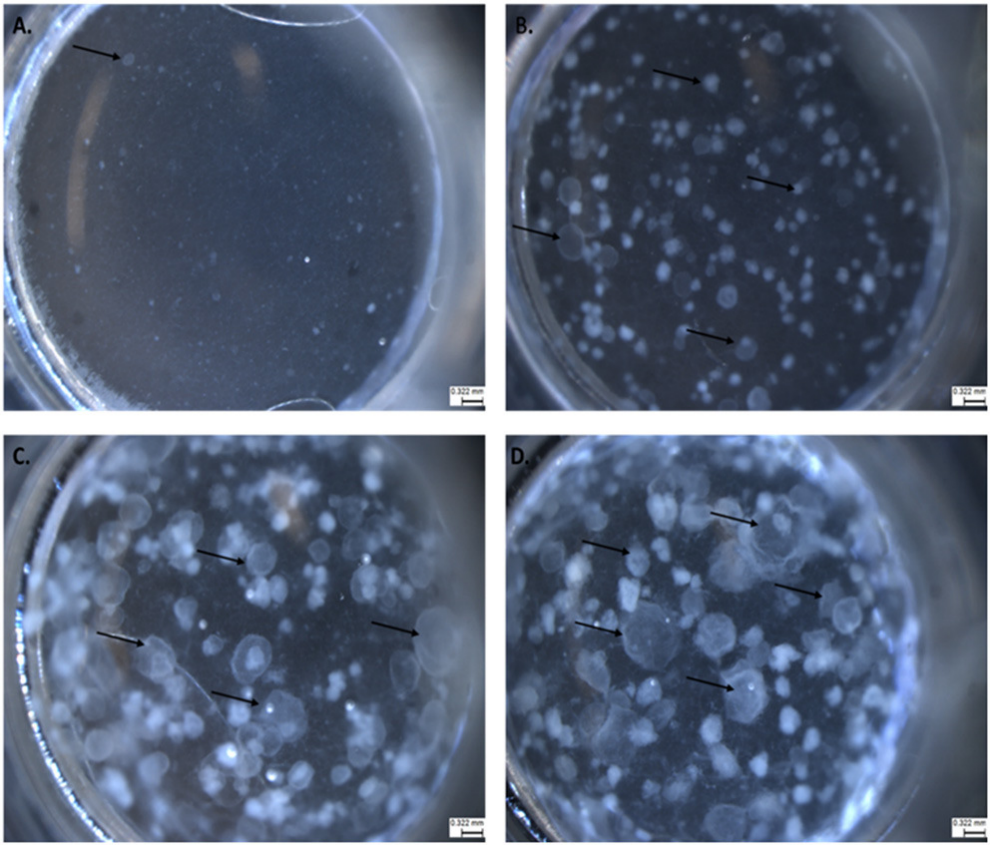
The progression of lung organoid development. Each black arrow indicates an organoid. **(A)** Initial plating of primary lung epithelial cells (EpCAM) and primary lung fibroblast cells (CD140a) as viewed under light microscopy. **(B)** The development of lung organoids within 0.5–1.5 weeks. **(C)** The development of lung organoids between 1.5 and 2 weeks. **(D)** The development of lung organoids between 2 and 5 weeks of growth. All scale bars are 0.322 mm.

**Figure 2:**
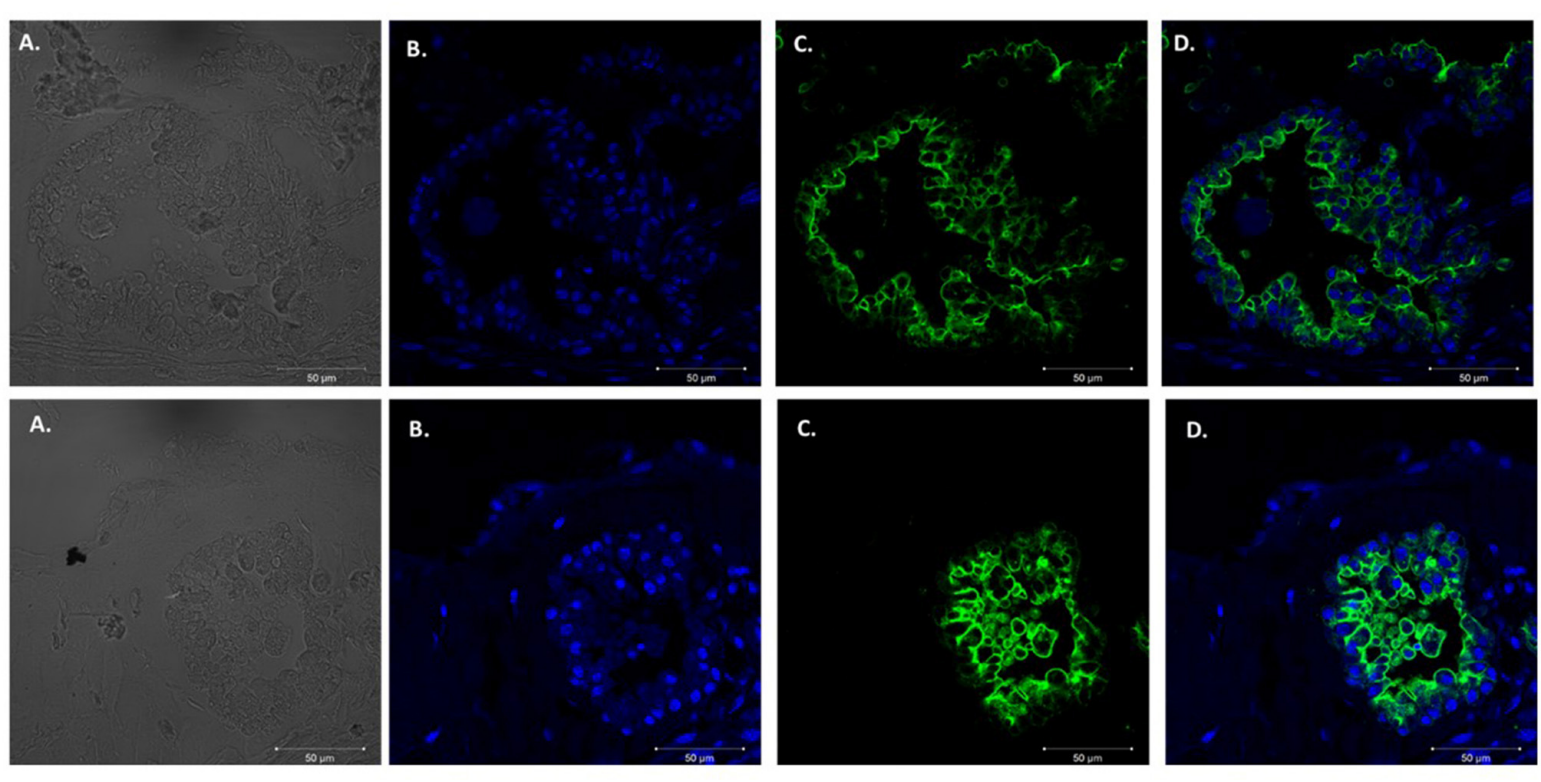
Type 1 alveolar cell marker expression. Two independent organoids stained with Aquaporin 5 antibodies (AQP5); a membrane bound cell marker for type I alveolar epithelial cells pseudo labeled green. Left to right. **(A)** Bright field images organoids. **(B)** Nuclei stained with DAPI. **(C)** AQP5 (membrane bound staining pattern) of AEC1 cells. **(D)** Combination of nuclei and AQP5 staining. Scale bars are 50 μm.

**Figure 3:**
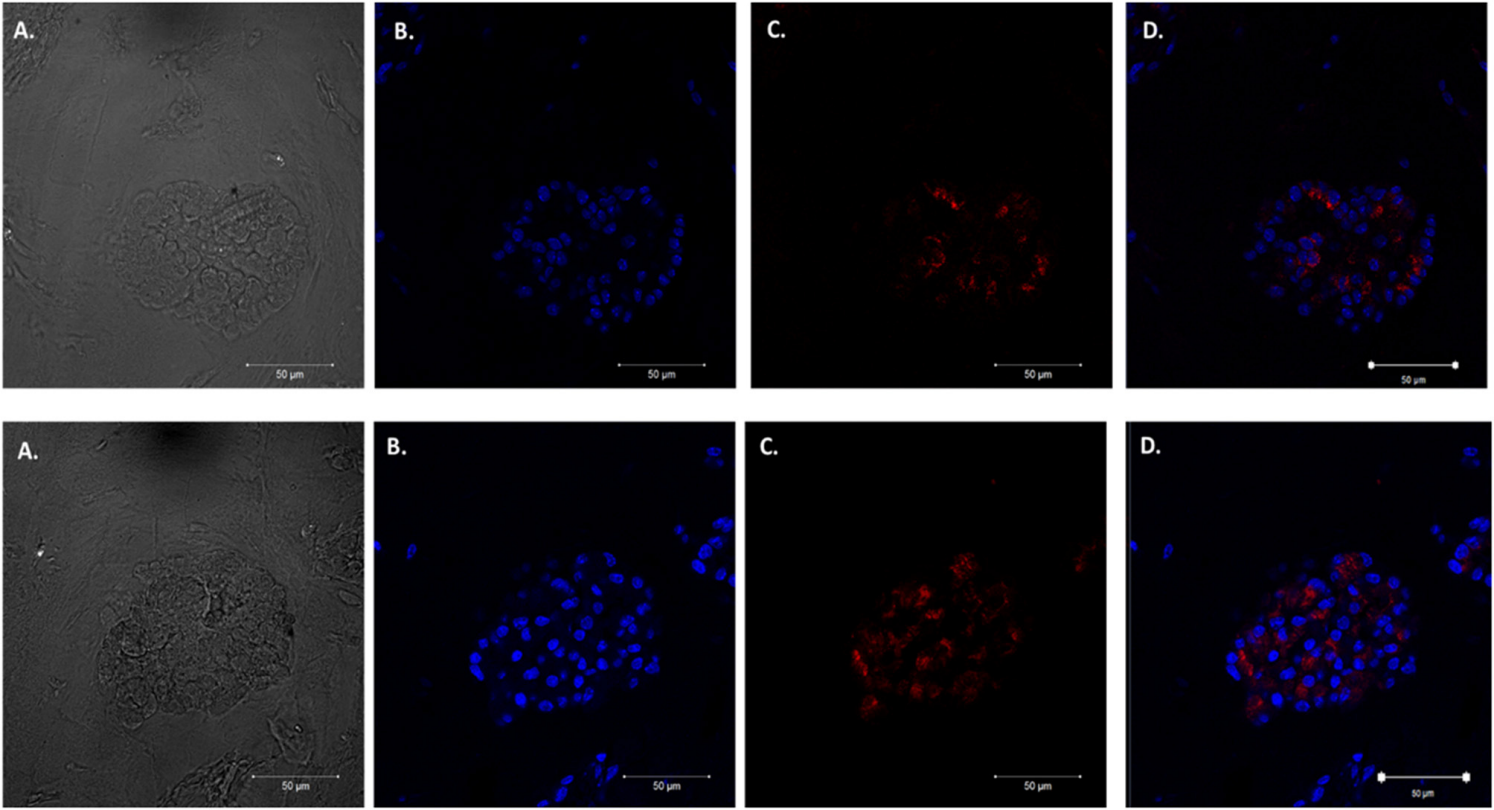
Type 2 alveolar cell marker expression. Two independent organoids labeled with Surfactant C antibodies (SFTPC), cytoplasmic cell marker for type II alveolar epithelial cells pseudo labeled red. Left to right. Two left panels display **(A)** Bright field images of organoids. **(B)** Nuclei stained with DAPI. **(C)** SFTPC (cytoplasmic staining pattern) for AEC2 cells. **(D)** Combination of DAPI nuclei and SFTPC staining. Scale bars are 50 μm.

**Figure 4:**
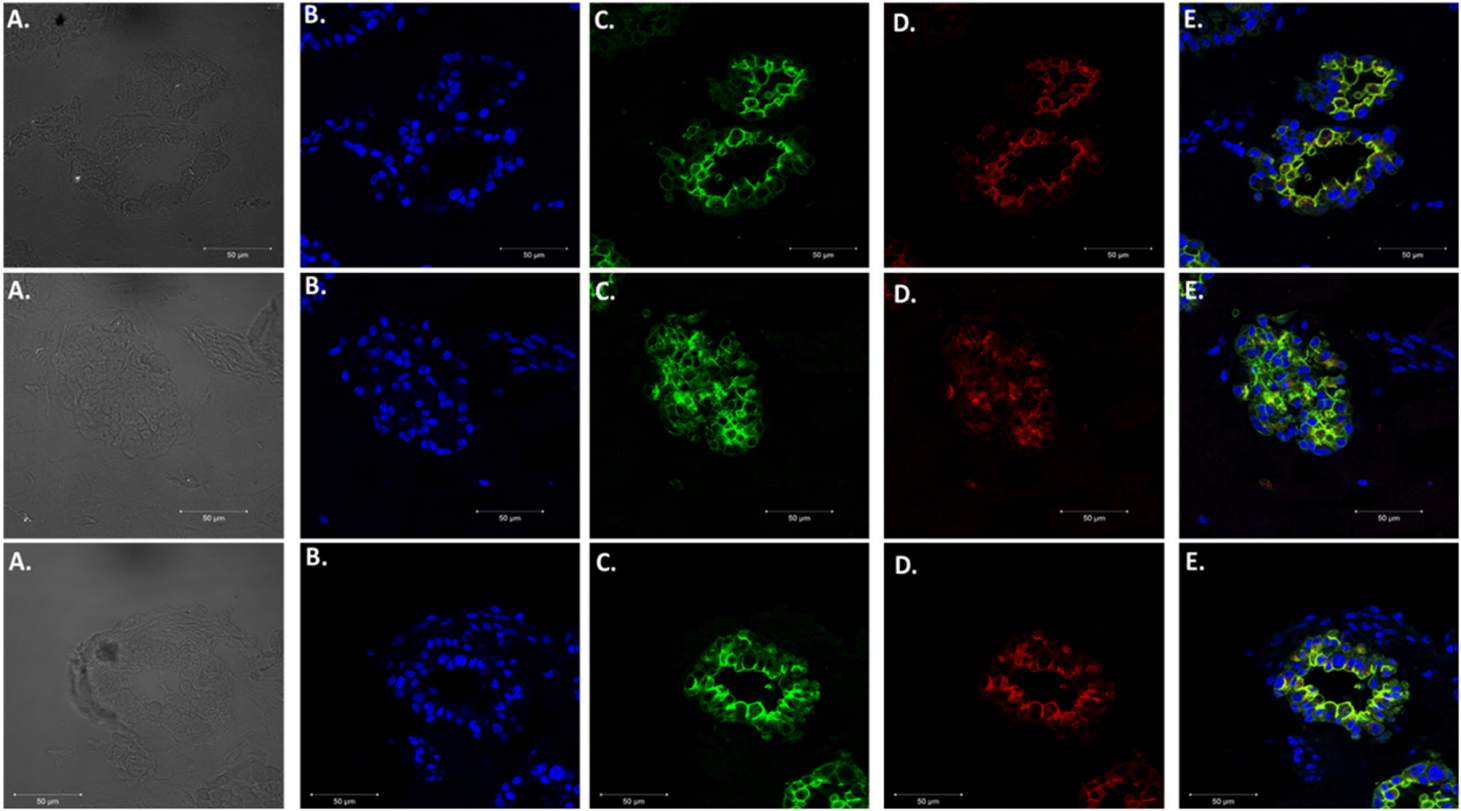
Expression of Type 1 and Type 2 alveolar epithelial cell markers. Three independent organoids stained with both cell markers AQP5 (green) and SFTPC (red). From left to right. **(A)** Bright field images of organoids. **(B)** Stained with DAPI. **(C)** AQP5 5 staining. **(D)** SFTPC staining. **(E)** Merged image of all cell markers. Scale bars are 50 μm.

We adapted techniques from previous studies illustrating that distal lung epithelial stem cells must be co-cultured with fibroblasts to proliferate and develop into organoids (McQualter et al., 2010; Barkauskas et al., 2013; Gokey et al., 2021). Thirty thousand epithelial cells were co-cultured with 100,000–150,000 fibroblasts per well. The progression of organoid development and growth over time under these culture conditions were tracked over time in culture using light microscopy (Figure 1). Each black arrow points to an organoid. Once we confirmed a reproducible technique of developing lung organoids, we evaluated the cell composition of the organoids, especially differentiation of AEC1. A previous study showed that culturing epithelial stem cells with fibroblasts can produce three different morphological organoids (Chen et al., 2012). As *Pneumocystis* spp. preferentially attaches to AEC1 and avoids AEC2 we aimed to establish organoids with AEC1 and some AEC2. A previous study showed that treating lung organoids with Rho-associated protein kinase inhibitor (ROCK-I) Y-27632, and Leukemia Inhibitory Factor (LIF) growth effectors alters the differentiation potential of organoids (Hegab et al., 2015). Treatment with ROCK-I resulted in unlimited propagation of epithelial cells in the presence of fibroblast feeder cells. Treatment of organoids with LIF increased the number of all three types of organoid colonies per well and significantly increased the number of organoids expressing AEC1 and AEC2 cell markers. Based on these data we treated organoid colonies with both effectors to achieve larger >100 um sized organoids that contained AEC1. To confirm the presence of AEC1 and AEC2 cells, organoids were sectioned and stained with Aquaporin 5 (AQP5) and Surfactant Protein -C (SFTPC) antibody markers for AEC1 and AEC2, respectively. Shown in Figures 2–4 are different organoids that expressed both markers, where AQP5 was pseudo labeled green and SFTPC was pseudo labeled red.

### Injection of Murine Lung Organoids With *P. murina*

**Figure 5:**
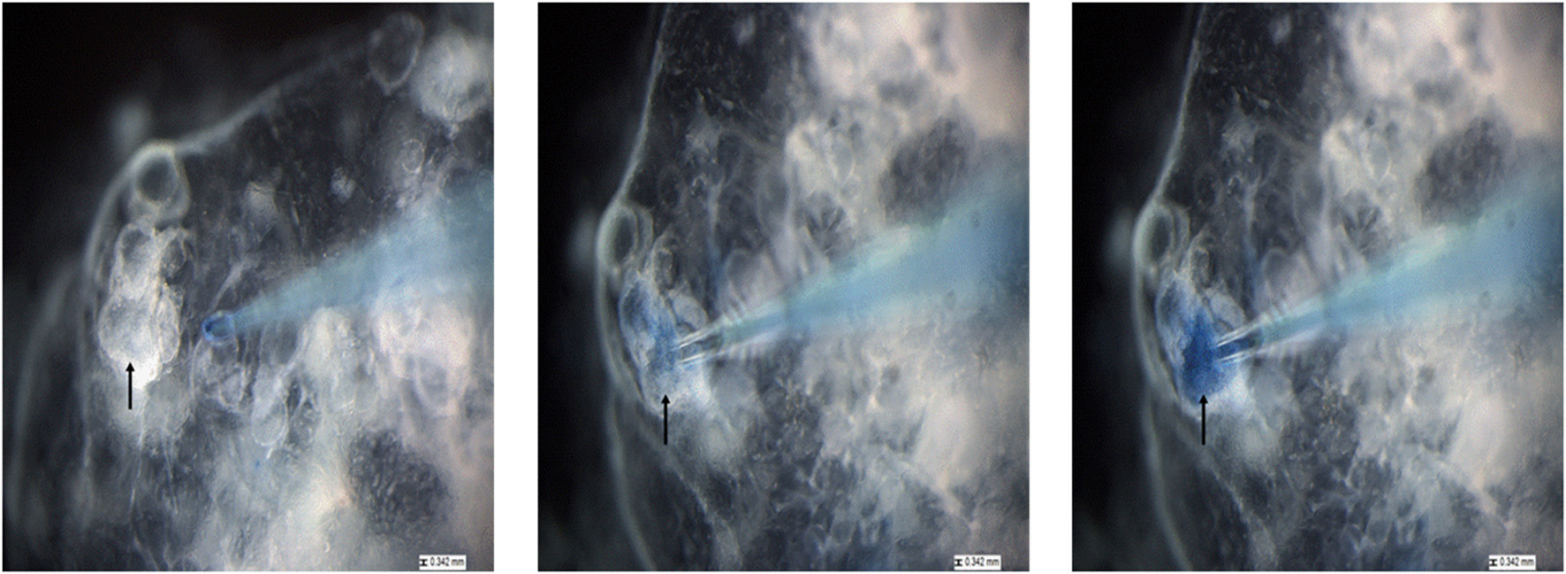
The process of injecting lung organoids with *P. murina* stained with trypan blue. Black arrow is pointing at the organoid that is injected with *P. murina*. After about 2 weeks of growth organoids are injected with *P. murina*. From left to right is a depiction of the process of injecting an organoid with *P. murina* stained with trypan blue. Scale bars for the images are 0.342 mm.

To allow visualization of successful injection, *P. murina* were treated with a 1% trypan blue solution and used for micro-injection. As shown in Figure 5, we targeted sizeable organoids (black arrow). With a micro-needle filled with stained *P. murina*, we showed that the murine lung organoids were penetrated and held injected *P. murina* without leakage. The lack of leakage was indicated by the lack of extra-organoid staining and sequestration within the organoids.

### Proof of Concept by Confirming the Presence of *P. murina* in Murine Lung Organoids

After confirming that these organoids were injectable, it was necessary to validate visualization of *P. murina* without prestaining with trypan blue. Prestaining organisms before injection may damage or prevent any growth for future experiments. To accomplish this, we injected unstained *P. murina* into organoids at the same density as described above. Immediately after injections, the organoids were fixed and stained with appropriate antibodies. To stain *P. murina*, we used rabbit anti-major surface glycoprotein antibodies (MSG). MSG is expressed on all life cycle stages of *Pneumocystis* spp. and for this reason, it is an excellent marker to detect *P. murina* organisms. AQP5 antibodies were used to stain AEC1 cells pseudo color green, and SFTPC antibodies were used to stained AEC2 cells pseudo-colored red.

**Figure 6:**
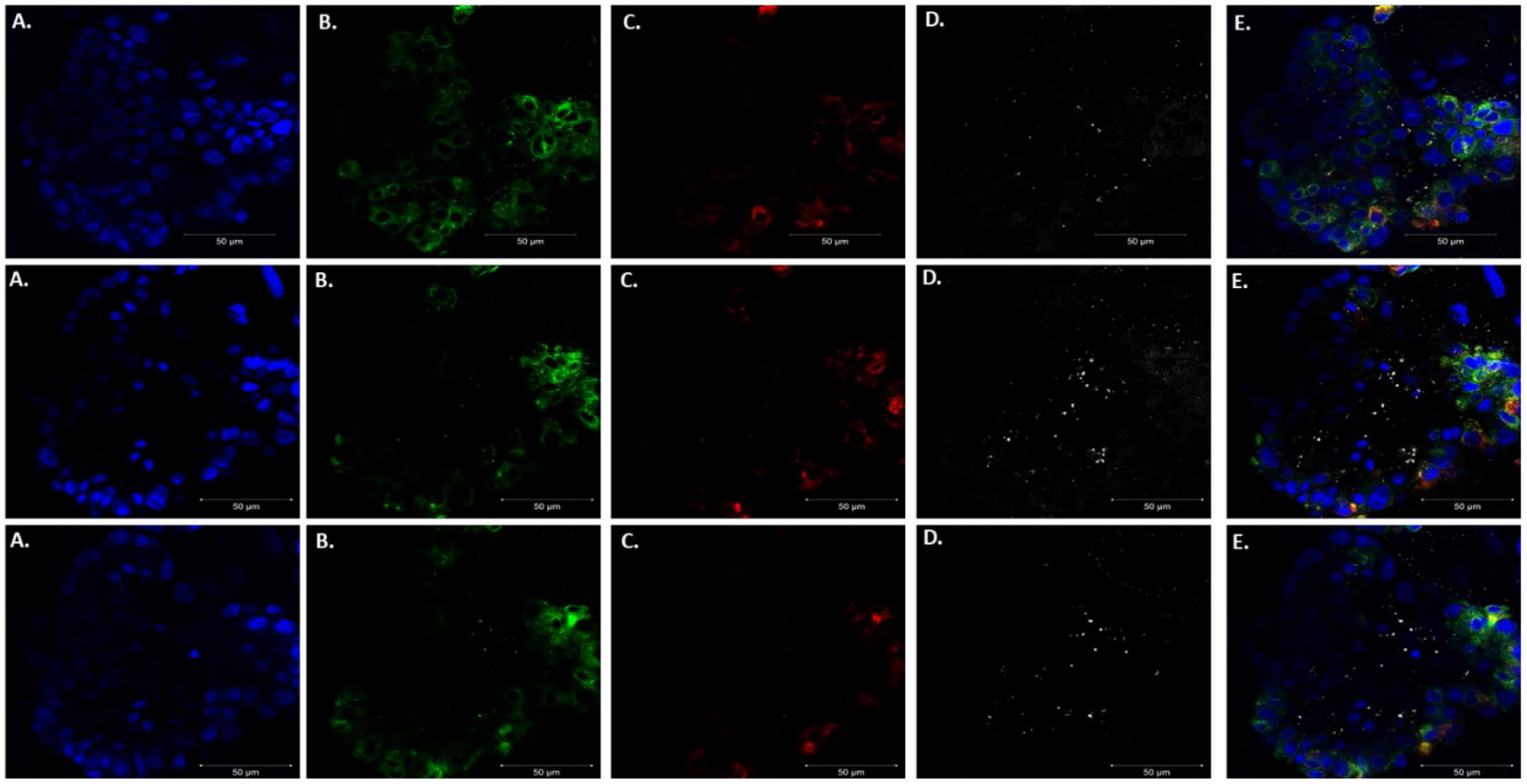
Injecting lung organoids with *P. murina*. Whole lung organoid Z stack images (top, middle and bottom images) of an organoid injected with *P. murina*. **(A)** Stained with DAPI for nuclear staining (blue). **(B)** AQP5 AEC1 cell membrane marker (green). **(C)** SFTPC AEC 2 cell cytoplasmic marker (red). **(D)** MSG staining *P. murina* organisms (white). **(E)** Merged image of all cell markers and *P. murina* staining. Scale bars for the images are 50 μm.

Figure 6 illustrates staining of whole-mount organoid injected with *P. murina*., stained with MSG pseudo-colored white. This visualization is proof that lung organoids can potentially be used to propagate and maintain *Pneumocystis* spp. Considering the requirement to undergo sexual reproduction, lung organoids may provide an ideal environment for expression of this mode of replication.

The goal of this study was to establish lung organoids as a potential system to propagate and sustain *Pneumocystis* spp. growth with anticipated future expansion to study its life cycle. As depicted in Figure 1, 4-weeks of organoid growth results in varying organoid sizes. We determined that success rates of injection were highest in lung organoids at an average of 100–200 um in size. Injecting organoids smaller than 100 um is challenging and problematic for two reasons: (1) the lumen of the organoid needs to be large enough to hold injected material without leakage; and (2) organoids of smaller sizes collapse or break open during injection attempts.

We are currently pursuing efforts to evaluate growth in this system, but wanted to introduce this proof of concept to the scientific community to demonstrate the potential use of lung organoids to study *Pneumocystis* spp. and provide access to this technology.

## Data Availability Statement

The original contributions presented in the study are included in the article/supplementary material, further inquiries can be directed to the corresponding author/s.

## Ethics Statement

The animal study was reviewed and approved by Cincinnati Veterans Medical Center, Instutional Animal Use and Care Committee.

## Author Contributions

MC, NT-M, A-KP, NW, and TP contributed to conception and design of the study. AA prepared the mice used in these studies. JG taught the organoid techniques to NT-M. NT-M wrote the first draft and collaborated with MC, A-KP, NW, and TP on the final version of the manuscript. All authors contributed to the article and approved the submitted version.

## Conflict of Interest

The authors declare that the research was conducted in the absence of any commercial or financial relationships that could be construed as a potential conflict of interest.

## Publisher's Note

All claims expressed in this article are solely those of the authors and do not necessarily represent those of their affiliated organizations, or those of the publisher, the editors and the reviewers. Any product that may be evaluated in this article, or claim that may be made by its manufacturer, is not guaranteed or endorsed by the publisher.
